# Be familiar with benign pediatric head and neck lesions! Image interpretation guides to overcome your weakness

**DOI:** 10.1007/s11604-024-01697-y

**Published:** 2024-12-04

**Authors:** Motoo Nakagawa, Wenya Zhao, Kumiko Nozawa, Noriko Aida, Norio Shiraki, Yuki Yasuda, Akio Hiwatashi

**Affiliations:** 1 Department of Radiology, Nagoya City University Graduate School of Medical Sciences, 1 Kawasumi, Mizuho-Cho, Mizuho-Ku, Nagoya, Japan; 2 Department of Radiology, Kanagawa Children’s Medical Center, 2-138-4 Mutsukawa, Minami-Ku, Yokohama, Kanagawa 232-8555 Japan; 3 Department of Radiology, Yokohama City University Graduate School of Medicine, 3-9 Fukuura, Kanazawa-Ku, Yokohama, 236-0004 Japan; 4 Department of Radiology, Nagoya City University West Medical Center, 1-1-1 Hirate-Cho, Kita-Ku, Nagoya, 462-8508 Japan

**Keywords:** Pediatric, Head and neck, Image findings, Benign lesions

## Abstract

Pediatric head and neck lesions include three main categories: congenital, inflammatory, and neoplastic. It is important for management to understand the imaging features. The purpose of this pictorial review is to demonstrate the imaging features of benign head and neck lesions of pediatric patients. To get tips on overcoming anxiety about this area, this article also presents pitfalls related to each of these diseases.

## Introduction

In Japan, there are few radiologists specialized in the pediatric disease. There are also few radiologists who are familiar with diseases of the head and neck region in children.

Mass lesions of the head and neck region in children can be divided into three main categories: congenital, inflammatory, and neoplastic. This article describes the imaging findings of benign lesions in the pediatric head and neck region for radiologists who do not specialize in pediatrics. It also describes the pitfalls that can easily be encountered when reading the imaging of each disease.

Ultrasound is a very useful imaging modality in pediatric head and neck disease because it does not use radiation and does not require sedation. Imaging should begin with ultrasound and other noninvasive tests, with CT and MRI added for more detailed evaluation as needed. For this reason, this paper presents some ultrasound images for each disease. We hope that you will become familiar with ultrasonography as well as pediatric radiology.

## Thyroglossal duct cyst

Thyroglossal duct cysts account for 70% of congenital cervical lesions. Most are found in children under 10 years of age. Most are found as painless masses, although bleeding or infection can cause pain.

The thyroid gland arises from the foramen cecum at the base of the tongue, descends in front of the hyoid bone and thyroid cartilage, and finally reaches a position anterior to the trachea at 7 weeks of gestation [1]. This descending tract of the thyroid gland is the thyroglossal duct, which usually regresses between 8 and 10 weeks of gestation. However, if a portion of the duct remains and the epithelium acquires secretory ability due to inflammation or other factors, a cystic lesion is formed. This is a thyroglossal duct cyst.

### Imaging findings

On ultrasound, thyroglossal duct cysts are seen as a thin-walled cystic lesion with low echogenicity. About half of these lesions are uniformly hypoechoic, while the other half show hyperechoic or heterogeneous echogenicity [2]. Echogenicity increases when the internal protein concentration is high, and a septum may be seen if infection is present previously.

**Fig. 1:**
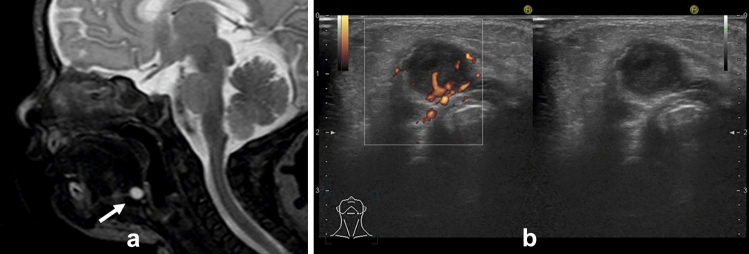
**a** One-year-old boy with the thyroglossal duct cyst. T2 weighted image incidentally revealed cyst which showed high signal intensity in front of the hyoid bone. **b** Two-year-old boy with the thyroglossal duct cyst. Ultrasonography revealed hypoechoic cyst in front of the hyoid bone. Echogenicity was slightly higher than simple cyst and flow was detected on Doppler ultrasonography due to infection of the thyroglossal duct cyst

Computed tomography (CT) and magnetic resonance imaging (MRI) show it as a thin-walled cystic mass with well-defined borders (Fig. 1a). The interior of the lesion is usually homogeneous and shows CT values and signal intensity like water. When complicated by inflammation or infection, wall thickening, and contrast enhancement are seen. In addition, CT may show slightly high CT value and T1-weighted image (WI) may show high signal intensity, reflecting protein content and hemorrhage in the cyst.

The differential diagnosis of thyroglossal duct cysts includes dermoid or epidermoid cysts and second gill cysts. Dermoid or epidermoid cysts can also be midline cystic neck masses. Thyroid hyoid cysts are often adjacent to the hyoid bone, which can be an important feature for differentiation. The second branchial cleft cysts are also an anterior cervical cystic mass. However, branchial cleft cysts are lateral and not adjacent to the hyoid bone.

### Pitfalls

Typically, thyroglossal duct cysts are commonly located in the median, but 38% were slightly off midline [2]. If this lesion is complicated by infection, the surrounding echogenicity may be elevated, and blood flow may be seen on Doppler ultrasonography (Fig. 1b).

## Ectopic thyroid gland

Ectopic thyroid is a condition in which thyroid tissue is found in an unusual location (anterior to the second to fourth tracheal cartilages) and generally occurs along the embryological descending route of the thyroid gland.

Ectopic thyroid can be divided into two types as follows. First type: where the thyroid tissue remains on the descending route of development, lacking the normal thyroid gland in usual area. The ectopic thyroid gland is the only thyroid gland. Second type: where the normal thyroid gland is in the normal site, but accessory thyroid tissue is also found on the descending route of development or in other sites. In rare cases, the normal thyroid gland is absent and ectopic thyroid tissue is present in two locations (dualectopy).

### Image findings

**Fig. 2:**
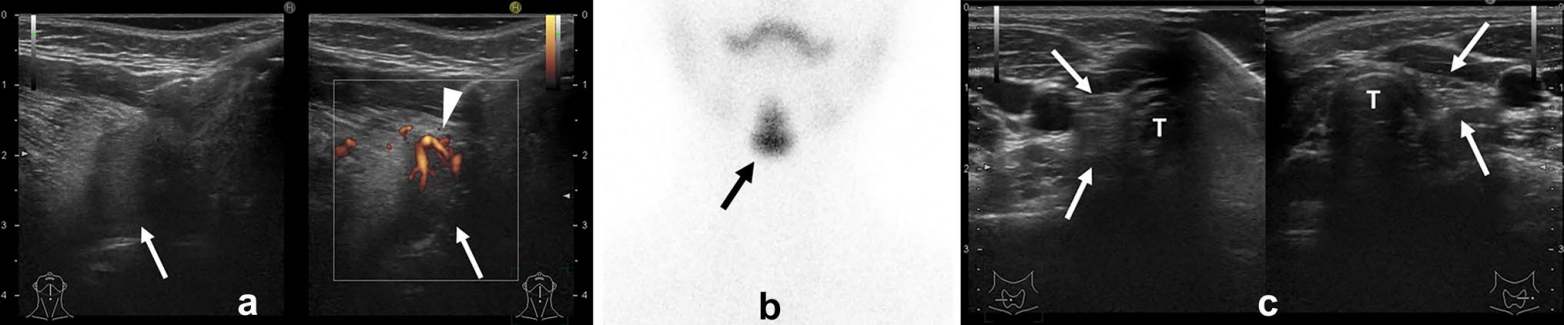
Five-year-old girl with ectopic thyroid gland. **a** Ultrasound showed a nodule with lower echogenicity than the surrounding tissue at the base of the tongue. Power Doppler showed blood flow in the nodule. **b** 99mTc-O4- Scintigraphy revealed uptake of lingual thyroid. There was no uptake of normal thyroid where should be. **c** Ultrasound showed slight hyper-echoic areas (arrows) where the thyroid gland should be. These slight hyper-echoic areas were considered as fat or interstitium in the dead space. Normal thyroid tissue was not able to be detected around there. T: trachea

The echogenicity of an ectopic thyroid gland can vary. On ultrasound, the normal thyroid gland is homogeneous with a slightly high echogenicity. The internal echogenicity of ectopic thyroid tissue is showed as isoechoic (similar echogenicity with adjacent tissue) or variable (Fig. 2a) [3].

CT and MRI also can show ectopic thymus as a well-defined nodule. Thyroid scintigraphy such as technetium-99 m pertechnetate are also useful because they show increased uptake in ectopic thyroid tissue (Fig. 2b).

### Pitfalls

Usually, cases have ectopic thyroid gland, the normal thyroid gland is not present where it should be. However, ultrasound shows a slight hyper-echoic area where the thyroid gland should be, despite the absence of normal thyroid tissue (Fig. 2c) [4]. This is likely due to the presence of fat and interstitium in the dead space. It should not be mistaken for hypoplasia or atrophy of the thyroid gland.

## Ectopic thymus

The thymus primarily originates from the ventral wing of the third pharyngeal pouch around the sixth week of gestation [1]. By the seventh week, the thymic primordia form the thymopharyngeal duct, descending caudally and medially to the anterior mediastinum. Fusion of thymic lobes occurs during the eighth week, positioning the thymus anterior superior to the heart and great vessels, while the thymopharyngeal duct retracts.

The thymus exhibits its most significant activity during childhood, continuously growing in size from the neonatal period through puberty, reaching its maximum volume during adolescence.

Thymic tissue primarily resides in the anterior mediastinum. Ectopic thymus refers to thymic tissue located outside the anterior mediastinum. Common ectopic locations include the anterior medial region of the sternocleidomastoid muscle and occasionally within the thyroid gland.

### Imaging findings

**Fig. 3:**
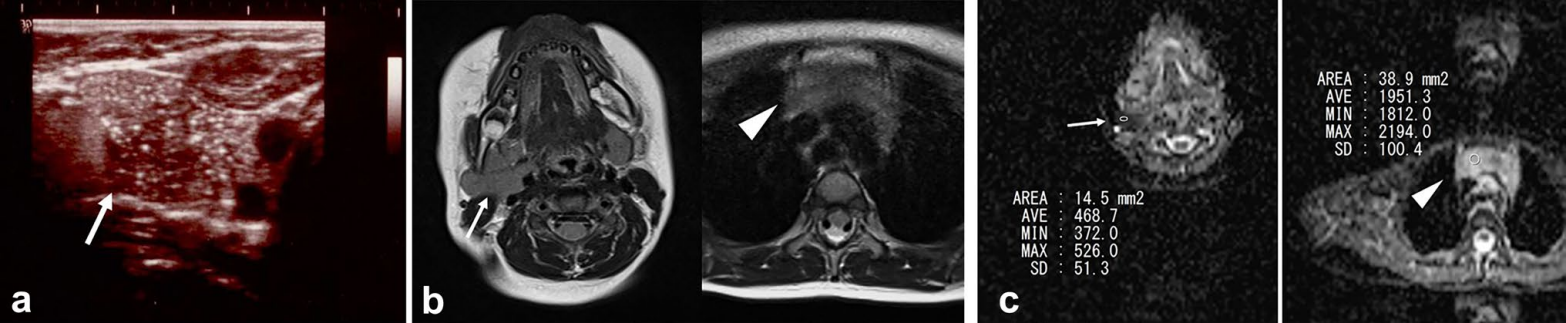
Two-year-old girl with ectopic thymus. **a** Ultrasonography revealed mass (arrow) at the right parapharyngeal space. The mass has scattered dots and lines of high echoic areas, which are known as “starry sky” or “dot and dash” appearance. **b** On T2 weighted image, mass (arrow) shows slightly high signal intensity which are same as that of normal mediastinal thymus (arrow head). **c** Apparent diffusion coefficient (ADC) value of mass (arrow: 468.7 x 10^−6^mm^2^/sec) was lower than that of normal mediastinal thymus (arrow head: 1951.3 x 10^−6^mm^2^/sec)

Ultrasonography reveals hypoechoic thymic tissue with multiple diffuse linear or branched hyperechoic foci, presenting a distinctive “starry sky” or “dot and dash” appearance (Fig. 3a) [5]. When ectopic thymus is within the thyroid gland, it exhibits a well-defined margin and a fusiform shape [6].

MRI demonstrates similar signal intensities between ectopic thymus in the cervical region and thymus in the regular region on T1WI and T2WI sequences (Fig. 3b) [7]. Additionally, MRI can show continuity between ectopic cervical thymus and mediastinal thymus in around 50% of cases.

### Pitfalls

On MRI, the cervical and mediastinal thymus show similar signal intensities in all sequences. However, apparent diffusion coefficient (ADC) values may differ between the two (Fig. 3c) [8]. It is unclear why the ADC values differ because no histological evaluation has been reported.

## Normal lymph nodes of pediatric neck

Lymph nodes with a short axis diameter of 5 mm or less are usually found in the neck of asymptomatic children. On cervical imaging studies, lymph nodes of children are depicted more numerous and larger than that of adults. It is necessary to be aware of the imaging findings of normal lymph nodes in children on imaging studies to avoid mistaking them for malignancy or lymph node metastases.

### Image findings

**Fig. 4:**
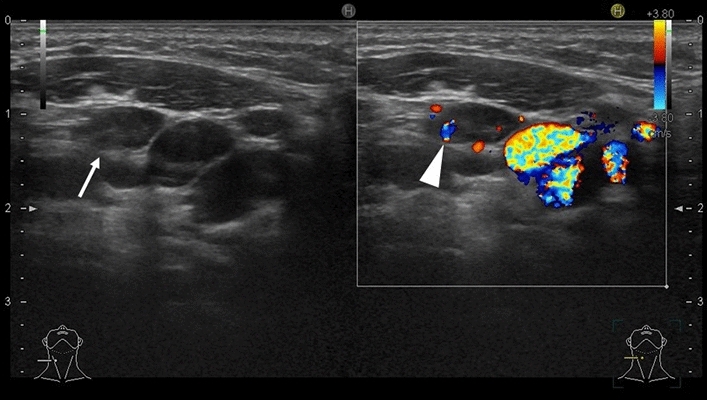
Ten-year-old girl had fever and palpable node at neck. Ultrasonography revealed low echoic nodule with linear high echogenicity (hilum) (arrow). Doppler US depicted flow at hilum of lymph node (arrow head). These are findings of normal lymph nodes.

On ultrasound, the normal lymph nodes are flat or oval hypoechogenic nodules with linear hyperechogenic hilum. In the normal neck, approximately 90% of lymph nodes with a maximum transverse diameter greater than 5 mm show echogenic hilus on ultrasonography (Fig. 4) [9]. Color Doppler imaging depicts blood flow to the hilum. Length to width is 2:1.

Infection or malignancy should be suspected when the lymph node has the following characteristics: (1) Large size (greater than 1.3 cm in long axis [10] or 1.2 cm in short axis [11], (2) long-to-short axis (L/S) ratio < 2, (3) heterogeneous echogenicity, (4) fusion to other lymph node, (5) irregular margin, (6) loss of hilum, (7) blood flow from areas other than the hilum [12].

### Pitfalls

Although there are various criteria to determine a lymph node as normal or abnormal on imaging, it must be judged comprehensively, not just by one factor.

## Kawasaki disease

Kawasaki disease, also called mucocutaneous lymph node syndrome (MCLS), is a systemic vasculitis of unknown cause that is common in infants under 4 years of age and has been on the increase in recent years. This disease should be treated as soon as possible because it can be complicated by coronary artery disease.

### Image findings

Cervical lymphadenitis, which has a similar clinical age of onset, is included in differential diagnosis. Compared to cervical lymphadenitis, Kawasaki disease tends to present as “clusters of grapes” or “bunches of grapes” with enlarged lymph nodes on ultrasonography [13]. There is no clear definition of a “group” or “cluster” on the images, and the diagnosis of a “grape cluster” or “cluster” appears to be subjective. The literature, which first reported this finding, states that “All the Kawasaki disease patients in our study had a single unilateral cervical mass. Transverse ultrasonograms demonstrated multiple enlarged nodes” [13]. Therefore, it is probably necessary to evaluate a set of “a single mass detected on palpation” and “a cluster of nodules detected on ultrasound”.

**Fig. 5:**
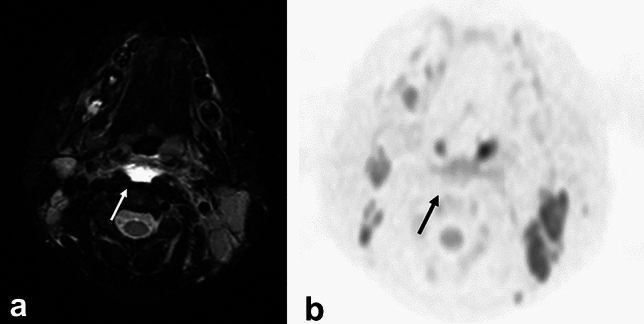
Ten-year-old girl with retropharyngeal edema due to Kawasaki disease. **a** T2 weighted image with fat suppression showed high intensity area at retropharyngeal space. **b** This area did not show as high intensity on diffusion weighted image

CT and MRI may show edematous changes in the retropharyngeal space (Fig. 5a) [14]. Unlike posterior pharyngeal abscesses, there is no contrast effect at the limbus on contrast-enhanced CT and MRI and no high-signal intensity on diffusion-weighted image (Fig. 5b).

Kawasaki disease is diagnosed based on clinical symptoms, but imaging evaluation may allow more appropriate diagnosis and treatment.

### Pitfalls

Kawasaki disease may be associated with edema in the lateral neck as well as in the retropharyngeal space [14]. Care should be taken not to confuse this with suppurative lymphadenitis or retropharyngeal abscess.

## Kikuchi disease (histiocytic necrotizing lymphadenitis)

Kikuchi’s disease (histiocytic necrotizing lymphadenitis) is a self-limited benign lymphadenitis of unknown cause that predominantly affects young women. This disease was first reported in 1972 by Kikuchi. Histopathologically, Kikuchi’s disease is characterized by cortical and paracortical necrosis with lymphocytic infiltration and absence of granulocytic infiltration.

### Image findings

**Fig. 6:**
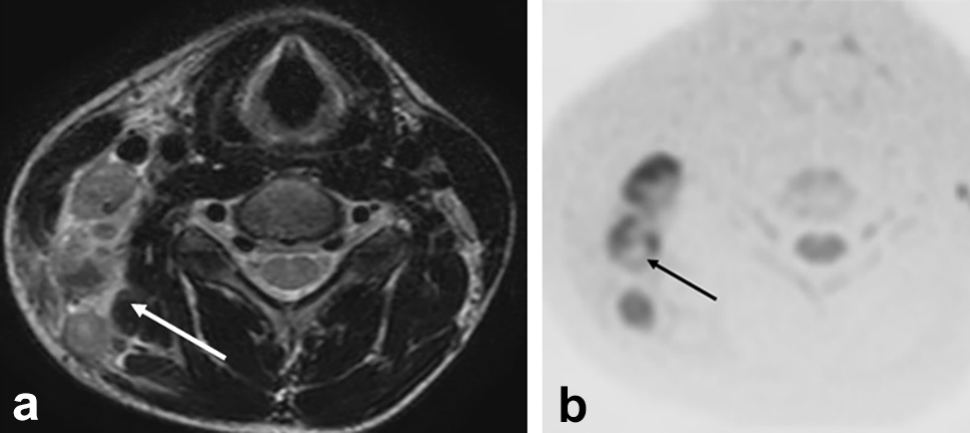
Twelve-year-old boy with Kikuchi disease (histiocytic necrotizing lymphadenitis). white blood cell: 2.5 x 10^3^/μL. **a** T2 weighted image revealed swelling lymph nodes with necrotic area which showed low-signal intensity (arrow). **b** Diffusion weighted image showed low intensity at the necrotic area

On ultrasonography, MRI, and contrast-enhanced CT, the lymph nodes in Kikuchi’s disease are enlarged and have internal necrosis. Usually, in diseases with necrosis or abscesses in their lymph nodes (pyogenic lymphadenitis, tuberculous lymphadenitis, cat scratch disease, lymph node metastasis, malignant lymphoma, Castleman’s disease, etc.), these necrotic areas show high signal on T2WI. However, in Kikuchi’s disease, necrotic areas show low signal intensity on T2WI (Fig. 6a) (a characteristic finding in Kikuchi’s disease) [15].

### Pitfalls

Necrosis of lymph nodes is characteristic of Kikuchi’s disease, but lymph nodes in Kikuchi’s disease do not necessarily have necrosis (17%) [16]. Therefore, the presence of necrosis should be confirmed carefully for each lymph node. It should also be noted that the absence of necrosis in the lymph nodes does not exclude Kikuchi disease.

## Retropharyngeal abscess and suppurative retropharyngeal lymphadenitis

The retropharyngeal space is a potential cavity that extends from the nasopharynx to the superior mediastinum. An abscess in this region is called a retropharyngeal abscess. The danger space is located behind the retropharyngeal space and formed by the sparse connective tissue between the pterygoid and prevertebral fascia, the inferior border of which extends to the mediastinum. If abscesses of the retropharyngeal space spread to the danger space, infection can reach the mediastinum and increase mortality.

Usually, in children younger than 5 years, upper respiratory tract infection precedes suppurative retropharyngeal lymphadenitis and eventually a retropharyngeal abscess. In older children and adults, trauma to the posterior pharynx may result in infection of the retropharyngeal space and abscess formation.

### Image findings

CT and MRI show fluid retention consistent with the retropharyngeal space. Contrast enhancement with contrast medium is seen at the margins of the lesion. Diffusion-weighted images show internal high signal intensity.

### Pitfalls

**Fig. 7:**
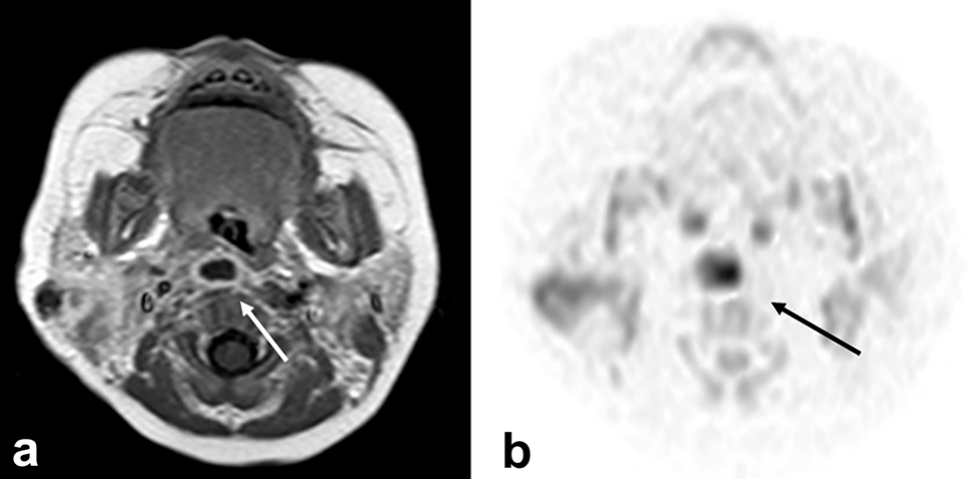
Eight-month-old boy with pyogenic retropharyngeal lymphadenitis. **a** Gadolinium enhanced T1 weighted image revealed cystic nodule with ring enhancement. Size was 12 x 7 mm. **b** Diffusion weighted image revealed high intensity at the cystic nodule (arrow). This lesion was not considered retropharyngeal abscess but pyogenic retropharyngeal lymphadenitis because this lesion contained by the nodal capsule. The lesion subsequently healed with conservative treatment with antibiotics

The preliminary stage of a retropharyngeal abscess is suppurative retropharyngeal lymphadenitis, which can perforate and spread to the retropharyngeal space to become a retropharyngeal abscess. Retropharyngeal abscess and retropharyngeal lymphadenitis are sometimes confused. In the case of a retropharyngeal abscess, incision and drainage are necessary. Retropharyngeal lymphadenitis, however, often improves with antibiotic therapy. Because of the difference in treatment strategy, some authors believe that these two conditions should be differentiated clearly [17]. Suppurative retropharyngeal lymphadenitis is contained by the nodal capsule (Fig. 7), while a retropharyngeal abscess is a suppurative collection within the retropharyngeal space.

Retropharyngeal edema is sometimes seen in Kawasaki disease and can be confused with a retropharyngeal abscess. Retropharyngeal edema in Kawasaki disease does not show a contrast effect at the margins of the lesion.

## Fibromatosis colli

Fibromatosis colli is a mass of well-differentiated fibroblasts and collagen fibers that arises within the sternocleidomastoid muscle. It is often detected clinically as cervical swelling. It reaches its maximum size at 2–3 weeks of age and then gradually shrinks. Rarely, there are cases that do not regress. In such cases, excision is considered because fibromatosis colli can cause torticollis. The cause of fibromatosis colli is thought to be fibrosis of the sternocleidomastoid muscle due to delivery trauma or venous insufficiency.

### Imaging findings

**Fig. 8:**
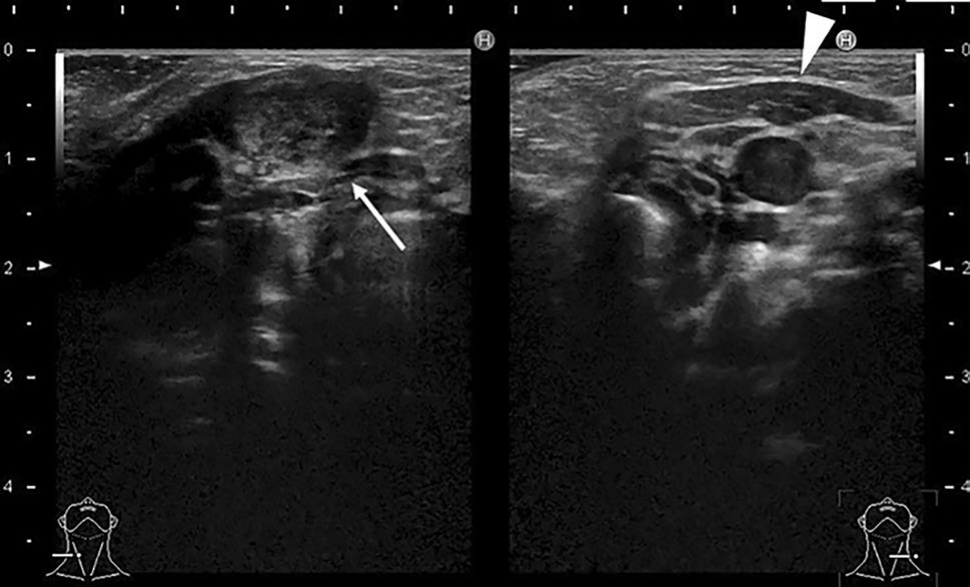
Two-month-old girl with hypothyroidism. Incidentally, fibromatosis colli of right sternocleidomastoid was detected. Ultrasonography revealed right sternocleidomastoid muscle (arrow) was thicker than that of left one (arrow head). Internal echogenicity of right sternocleidomastoid was high

Ultrasonography reveals fibromatosis colli as a spindle-shaped thickening confined to the sternocleidomastoid muscle. Internal echogenicity varies from low to high (Fig. 8) [18]. On MRI, the entire mass is pale and high signal on T2-weighted images. Low-signal areas may be mixed, reflecting the fibrous component within the tumor.

Malignant tumors such as rhabdomyosarcoma, neuroblastoma, and fibrosarcoma are given as differential diagnosis. However, fibromatosis colli is by far the most common tumor seen in the sternocleidomastoid muscle in the neonatal period than these malignant diseases.

### Pitfalls

Because fibromatosis colli is generally diagnosed clinically, imaging studies are requested infrequently. It is possible that fibromatosis colli may be coincidentally found in the sternocleidomastoid muscle when imaging studies of the neck are performed on a neonate for other disease. Therefore, this disease and its imaging findings should be recognized.

## Lymphatic malformations

Lymphangitic malformations are classified as vascular malformations in “International Society for the Study of Vascular Anomalies (ISSVA) classification.” The term “lymphangioma” has also been used, but since the pathology is essentially dysplastic rather than neoplastic, the name “lymphatic malformation” is considered more appropriate. Although they can occur at any site, they are particularly common in the head and neck region. Lymphoid malformations account for about half of all cases in the head and neck, where lymphatic tissue is well developed [19]. Lymphangitic malformations are classified into macrocystic type, microcystic type, and mixed type according to the size of cysts in the ISSVA classification. Generalized lymphatic anomaly that extends to multiple organs and Gorham’s disease that presents with lytic bone are other lymphatic malformations, but they are quite rare.

### Image findings

The first imaging approach is ultrasonography. In the macrocystic type, ultrasonography reveals lesion as anechogenic area with septal septa, sometimes with partial hyperechoic changes or fluid formation due to hemorrhage or infection. In the microcystic type, the entire lymphangioleiomyomatosis may be seen as hyperechoic lesions due to the presence of numerous microcysts [20].

### Pitfalls

**Fig. 9:**
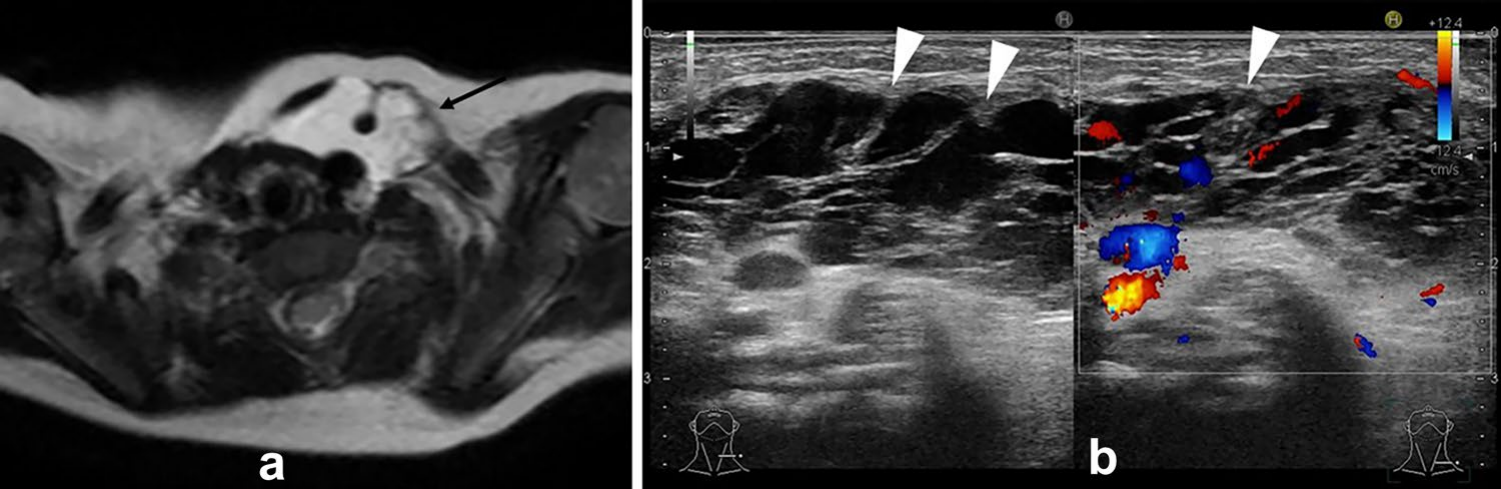
Three-month-old boy with lymphatic malformation. **a** T2 weighted image showed cystic lesion with high intensity (arrow) at left side of neck. **b** Ultrasonography showed cystic lesion with low echogenicity and some septa (arrow heads). The septa of this lesion were depicted clearer on ultrasonography than that of T2 weighted image

Sclerotherapy is one of the treatment modalities. Because the effect of sclerotherapy is small when the cyst is small, it is necessary to classify the cysts into macrocystic and microcystic types in deciding the treatment strategy. Some literature considers a cyst to be macrocystic if it is 2 cm or larger [21], but the criteria may vary from literature to literature. On imaging studies, the septum of the lymphatic malformation may differ in clarity between MRI and ultrasound (Fig. 9). This should be noted when assessing the size of the cyst.

## Hemangioma and vascular malformation

Abnormalities of the vascular system were divided into two categories in the ISSVA classification: vascular tumors due to neoplastic proliferation of cells and vascular malformations due to abnormal vascular morphology. Vascular tumors include infantile hemangiomas and congenital hemangiomas. Vascular malformations include lymphatic malformations and venous malformations.

Infantile hemangiomas are benign tumors in which glucose transporter-1 (GLUT-1)-positive capillary endothelial cells proliferate. Kaposiform hemangioendothelioma is also caused by proliferation of vascular endothelial cells. Kaposiform hemangioendothelioma is GLUT-1 negative [22].

Congenital hemangiomas are already present at birth. Depending on the course of the disease, congenital hemangiomas can be classified as rapid involuting congenital hemangiomas (RICH), non-involuting congenital hemangiomas (NICH), or partially involuting hemangiomas (PICH). (non-involuting congenital hemangioma), and PICH (partially involuting hemangioma). Congenital hemangiomas are GLUT-1 negative [23].

### Imaging findings

**Fig. 10:**
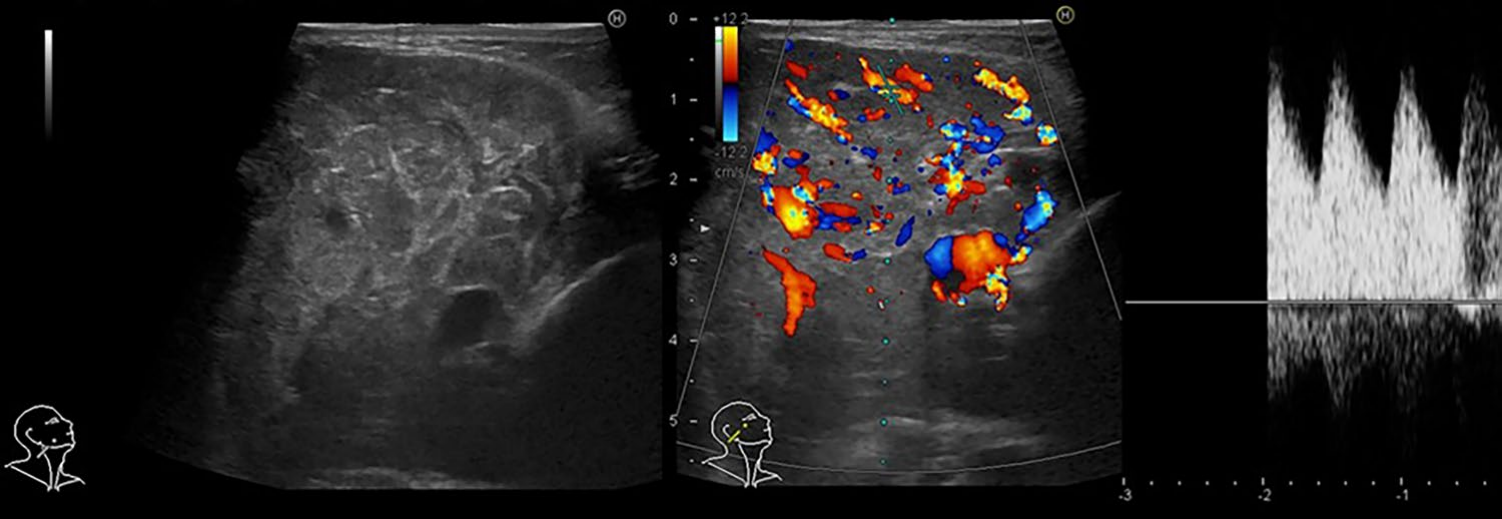
Four-month-old girl with infantile hemangioma. On ultrasonography, subcutaneous mass on right neck was revealed as a well-defined mass showing variable echogenicity and color Doppler shows arterial flow

On ultrasonography, an infantile hemangioma usually appears as a well-defined mass showing variable echogenicity and color Doppler shows increased internal vascular flow (Fig. 10). Compared to infantile hemangiomas, congenital hemangiomas often present as masses with a mixture of low- and high-intensity components, making it easier to delineate vascular structures on B-mode images [24].

MRI is also useful as a means of evaluating deeply located cases and their relationship to surrounding organs.

### Pitfalls

In some fields, lesions classified as vascular malformations in the ISVVA classification are sometimes referred to as “hemangiomas”. In children, hemangiomas and vascular malformations have different courses and prognoses, and should be diagnosed according to the ISSVA classification.

## Branchial cleft cyst

Around 4 weeks of gestation, a ridge known as the “branchial arch” develops. The branchial arch consists of a pair of branchial clefts (groove) and branchial pouch. Branchial cleft cysts are caused by the remnants of these branchial clefts. The lesions can occur as a cyst as well as a sinus or a fistula. Branchial cleft cysts are present from the first to fourth branchial clefts, with the second cleft being by far the most common.

Lesions originating from the first branchial cleft are located from the submandibular region to the external auditory canal, while those from the second branchial cleft occur between the lower 1/3 of the anterior margin of the sternocleidomastoid muscle and the palatine tonsils. Lesions originating from the third and fourth branchial clefts, both of which are associated with a pathway from near the upper pole of the thyroid gland to the pisiform fossa, are associated with fistula and cysts. Pyriform sinus fistula also occurs with abnormalities of the third and fourth branchial pouch [25].

### Imaging findings

**Fig. 11:**
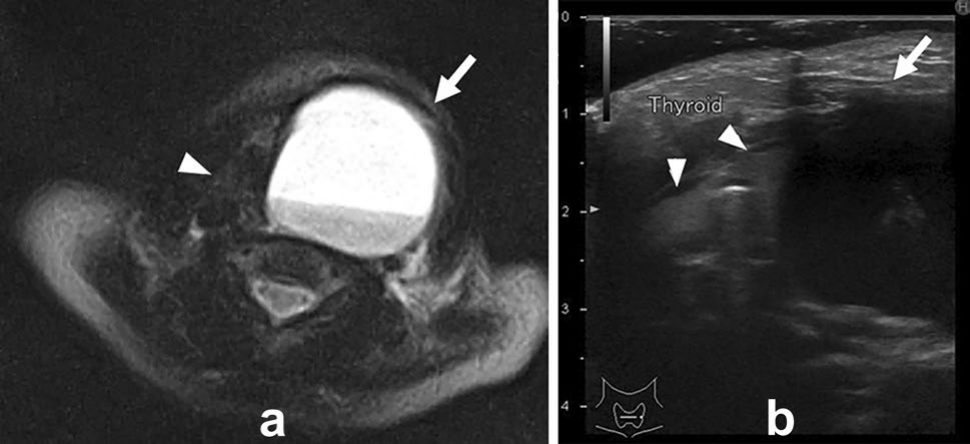
Ten-day-old girl with pyriform fistula (forth brachial cleft cyst) T2 weighted image **a** and ultrasonography **b** showed a cystic lesion (arrows) on the lateral side of the left lobe of the thyroid (arrow heads)

Ultrasonography is used to evaluate the nature of the cyst and the presence or absence of a fistula. Contrast-enhanced CT and MRI are also useful to clearly distinguish the relationship of the cyst to the surrounding tissues (Fig. 11). Differential diagnosis includes lymphangitic malformations and cervical cystic masses such as hemangiomas and ranula. The cyst is differentiated from a branchial cleft cyst by presence of blood flow, and form of distribution.

### Pitfalls

If a pyriform sinus fistula is suspected, a hypopharyngeal esophagogram should be considered to determine the presence or absence of a pisiform fistula because the fistula of a pyriform sinus fistula is sometimes difficult to detect on ultrasound, CT, or MRI [26].

## Conclusion

The imaging findings of benign head and neck lesions in children were reviewed. We hope that this paper could help you for future imaging studies in this area.
